# Influence of prior knowledge and experience on willingness to pay for home hospice services: a contingent valuation study

**DOI:** 10.1007/s10754-025-09393-8

**Published:** 2025-03-25

**Authors:** Caroline Steigenberger, Andrea M. Leiter, Uwe Siebert, Claudia Schusterschitz, Magdalena Flatscher-Thoeni

**Affiliations:** 1 Institute of Public Health, Medical Decision Making and Health Technology Assessment, Department of Public Health, Health Services Research and Health Technology Assessment, UMIT TIROL - University for Health Sciences and Technology, Hall in Tirol, Austria; 2 Department of Economics, Faculty of Economics and Statistics, University of Innsbruck, Innsbruck, Austria; 3 Division of Health Technology Assessment, ONCOTYROL - Center for Personalized Cancer Medicine, Innsbruck, Austria; 4 Institute for Technology Assessment, Department of Radiology, Massachusetts General Hospital, Harvard Medical School, Boston, MA USA; 5 Center for Health Decision Science, Departments of Epidemiology and Health Policy and Management, Harvard T.H. Chan School of Public Health, Boston, MA USA; 6 Institute of Psychology and Sports Medicine, Department of Psychology and Medical Science, UMIT TIROL - University for Health Sciences and Technology, Hall in Tirol, Austria

**Keywords:** Hospices, Terminal care, Attitude to death, Knowledge, Contingent valuation, Willingness to pay, D61, I18

## Abstract

Home hospice services contribute to dying in dignity by addressing medical and social needs at the end of life. The respective monetary valuation in a sense of willingness to pay is not available yet. We aim to quantify the benefits of home hospice services to society using society’s monetary valuation and examine the influence of prior knowledge and experience on willingness to pay for home hospice services. A nationwide cross-sectional contingent valuation study was conducted in Austria. We analyzed the impact of the determinants of interest on having a positive willingness to pay for home hospice services via multivariate Probit regression. Stated willingness to pay was analyzed using interval regression. Variable selection of potential influence factors and confounders was based on the literature. The variables of interest, prior knowledge of and experience with home hospice services, were represented by twelve related variables. We included 1262 respondents in the analysis. The two-part regression analysis showed a statistically significant positive impact on the probability of having a positive willingness to pay by prior knowledge of home hospice services, prior donations, and the wish of not dying alone. Prior donations also increase the level of willingness to pay. The probability of a positive willingness to pay was statistically significantly lower for respondents that stated to have experienced the death of more than ten close persons and perceived spending time with dying persons as burden than their respective counterparts. Our study provides evidence that information campaigns to increase the recognition and awareness of existing home hospice services could increase their perceived value in society.

**Supplementary Information:**

The online version contains supplementary material available at 10.1007/s10754-025-09393-8.

## Background

For many individuals, the preferred place of death is at home (Fereidouni et al., 2021). The same holds true when relatives are asked about the preferred place of death of their loved ones (Pinto et al., 2024). Therefore, congruence between the preferred and the actual place of death is a quality indicator for end-of-life care (García-Sanjuán et al., 2021). Home hospice teams support caregivers and patients and aim to meet the emotional and social needs of patients and their relatives (de Graaf et al., 2016). They support families in daily activities, with symptom management, and provide bereavement support at the end of life (de Graaf et al., 2016; Hui et al., 2013) (see Online Resource 1).

In addition, home hospice services lead to increased patient satisfaction with care and a more efficient resource allocation if patients can be discharged from inpatient settings more quickly after acutely needed medical care has been provided (Hudson et al., 2019). In a study by Kumar et al. (2017), cancer patients’ families were interviewed after patients’ death on their experiences with hospice care. The main findings indicated that hospice care is associated with improved symptom management and the achievement of patient-centered goals, such as honoring patients' wishes and facilitating death at their preferred location. Additionally, hospice care contributes to a high quality of end-of-life care (Kumar et al., 2017). Promoting earlier and more frequent hospice enrollment could potentially improve the experience of cancer patients and their families at the end of life (Kumar et al., 2017). This issue is of global relevance, as the World Health Organization's World Health Assembly in 2021 emphasized the importance of meeting psychosocial or spiritual needs in addition to the ethical responsibility of health care systems to alleviate pain and suffering at the end of life (World Health Assembly, 2014). Enabling dying in dignity thus is an important issue for health policy makers to address (Fereidouni et al., 2021; Gatterer, 1999). However, little is known about how society monetarily evaluates home hospice services. While there are studies that use dichotomous choice experiments to determine individuals' willingness to pay for long-term care attributes (Amilon et al., 2020) and institutionalization aversion (Costa-Font, 2017). However, home hospice services are inherently different and therefore not directly comparable.

Focusing on society’s preferences for home hospice services, which describes the demand side, enables a comprehensive evaluation of the tangible and intangible aspects of this non-market good. However, when it comes to the comprehensive economic evaluation of hospice services, little empirical evidence is provided by the literature (Kim et al., 2011; Sueki, 2017). To fill this gap for home hospice services, we provide results on the monetized societal value of home hospice services by conducting a contingent valuation study to elicit societies’ hypothetical willingness to pay for home hospice services. With this study, we follow a well-established approach for the economic evaluation of non-market goods (Johnston et al., 2017).

We pay particular attention if this willingness to pay is influenced by prior knowledge of (home) hospice services and experience with home hospice services (as defined in Table 5), referring to the systematic review of Steigenberger et al. (2022). The aim of our analysis is to investigate the causal influence (Hernán, 2018) of prior knowledge and experience on the willingness to pay for home hospice services in the Austrian population and to determine the average willingness to pay per month for access to home hospice services when needed. A more detailed description of home hospice services in Austria is provided in Online Resource ESM 1.

## Methods

### Design

A cross-sectional study was performed applying a contingent valuation approach (O'Brien & Gafni, 1996) using double-bounded dichotomous choice questions (Hanemann, 1984; Hanemann & Loomis, 1991) to elicit Austrian societies’ preferences towards home hospice services in a telephone survey. Contingent valuation is a method for estimating the value that people place on goods or services for which there is no market price. It involves asking people directly how much they would be willing to pay for the goods or services in question. This hypothetical willingness to pay for a service is one way to estimate the value of the service.

The questionnaire for the interviews was designed by three research team members (AML, CSch, MFT) based on literature, expert interviews, and pilots to identify missing items and a reasonable range for the amounts of the bids. The hypothetical scenario refers to the question, “Assume that the public funds are no longer available and that the funding is to be provided by membership fees respectively earmarked contributions in the future, what would you be willing to pay as a monthly insurance contribution if public payments are canceled?” (see question 13 in the questionnaire in ESM 2). The questionnaire contained general questions on prior knowledge about hospice services, the respondents’ social environment and volunteering, fundamental values, their attitude towards and experience with death and dying, and sociodemographic characteristics. Prior to the contingent valuation question on willingness to pay, the interviewer explained that hospice services are provided to seriously ill, dying people and their relatives either in facilities such as hospitals or through mobile hospice teams providing hospice services directly in people's homes. Services include bereavement support, end-of-life care, support in everyday activities, or social support as conducting conversations. Respondents were also told that at the time of the survey approximately 2800 people provided home hospice services in more than 300,000 h and the costs related to the provision of home hospice services have been provided by public funds. The translated but not validated English version of the questionnaire can be accessed in Online Resource 2 (ESM 2).

For the contingent valuation questions on willingness to pay, participants were divided into two different settings, both based on the assumption that public funding would be withdrawn, and home hospice services would have to be financed differently in the future. In both variants respondents were informed that the intervention assessed was home hospice services provided in the household of the person cared for. In variant 1, participants were offered home hospice services as a club good. This means that anyone can voluntarily become a member of the hospice and, with active membership, use the services of mobile hospice teams at no additional cost. Non-members must find another solution for the assistance they require. In variant 2, home hospice services would be a public good and would be financed by dedicated contributions. This means that every person whose income is above the minimum subsistence level must make a contribution and in return can claim home hospice services for themselves. For humanitarian reasons, people who do not pay contributions can also make use of the services.

**Fig. 1:**
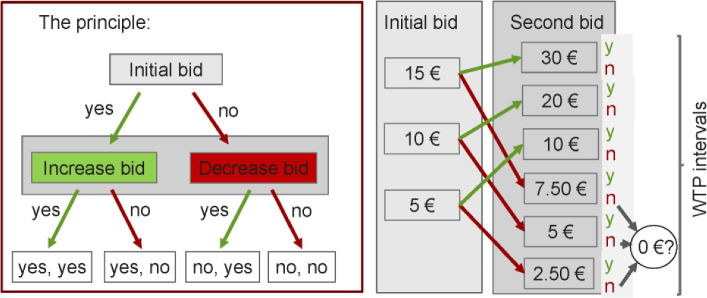
Structure of the contingent valuation question. Individuals were asked to answer two linked yes–no-questions, first, the initial bid, which is doubled if the answer is yes (green arrows) and halved if the answer is no (red arrows), and a second bid. Three different initial bids were used: 5, 10, and 15 euros per month. *Y* yes, *N* no, *WTP* willingness to pay (Color figure online)

In the next step, respondents were directly asked for their willingness to pay a monthly fee to access home hospice services in case of need. In a pretest with 59 respondents, we used open-ended questions for the willingness to pay to determine reasonable levels for the initial bids. Based on this pretest three initial bids were used: 5, 10, and 15 euros per month. The double-bounded dichotomous choice method means the respondents are asked two bidding questions, as shown in Fig. 1. The bidding structure, in which the price doubles or halves from the first bid to the second bid, enables easy responding and a high response rate.

### Population of interest

The target population of the study is the Austrian population (8.4 million in 2010).

A sample representative of the Austrian population (N = 1262) evaluated willingness to pay from an ex-ante perspective (Statistik Austria, 2021; Eurostat, 2021).

### Recruitment and data collection

Participants were recruited via telephone directly before the interview. Households randomly selected by the computer were called, and either the person whose birthday was next or who was the youngest was asked to participate in the interview among those in the household over 18 years of age. Questionnaire-based telephone interviews were conducted by trained interviewers from a market research institute in June and July 2010.

### Data management

For data management and analysis, we used the software Stata 17 (StataCorp, 2017). The data were entered by the market research institute that conducted the telephone interviews. K5-anonymity (Sweeney, 2002), was ensured during data analysis which means that no subgroup included less than five respondents. All variables were checked for plausibility, consistency, and missing data. Imputation was only necessary for “net income”, with approximately 25% missing values. We replaced the missing values with the imputed estimates of an ordered Probit model using net income as the dependent variable and socioeconomic characteristics as determinants.

### Variable definition and selection

Following good practice, variable selection for our research question was based on a comprehensive systematic literature review (Steigenberger et al., 2022) and additional scientific literature on potential causal relationships between the determinants and willingness to pay (see Table 5 in the Appendix). As described above, the questionnaire was designed in 2010 based on a comprehensive literature search and several expert interviews. The results of the systematic review from 2022 were used as an empirical basis for deciding which variables from this data set should be included into the empirical model. The literature review showed that in the studies included in the systematic review, variables related to the respondents’ awareness for and familiarity with the health service being assessed and prior involvement, including personal proximity to the health service assessed, play a role in evaluating healthcare services. However, few studies (e.g., Tran et al., 2018) addressed this aspect, and none was in the field of home hospice services. Factors identified as highly relevant within the systematic review were chosen as potential determinants of the willingness to pay for home hospice services. Determinants of interest related to prior knowledge of home hospice services were *stated knowledge of hospice*, *volunteering in hospice*, or having *donated to hospice* during the last years. Determinants of interest related to experience with home hospice services. were structured in three levels. The core variable on the first level was the *prior use of hospice* services by a person in the respondent’s household. On the second level, we put experiences related to death, which may or may not be related to hospice, comprising *experienced death* and the *number of close persons who died*. On the third level of experience, we group determinants on attitudes toward death and dying, expressed by the *wish not to die alone*, the *fear of death*, and the *perceived burden of spending time with terminally ill persons*. Descriptions of each variable can be found in Table 5 in the Appendix. We checked all variables for multi-collinearity using Spearman’s correlation coefficient. When more than one (partially) redundant variable was available for a specific thematic area, the variable with better data coverage was used.

### Statistical analysis

To check for plausibility and representativeness of our sample, the socioeconomic variables were compared with census data of the Austrian population (Statistik Austria, 2021; Eurostat, 2021). A two-part model (Cameron & Trivedi, 2005) was used to estimate the individuals’ willingness to pay. In the first part, we examined the probability of a positive willingness to pay, applying a multivariable Probit model in which the dependent variable is a binary variable that equals 1 for responses stating a positive willingness to pay and 0 otherwise. In the second part, we applied a multivariable interval regression, in which the dependent variables were the lower and the upper bound of the individual’s willingness to pay. To cover the non-negative willingness-to-pay range, we assumed that the latent willingness to pay is log-normally distributed. Standard errors of the estimates are clustered at the federal state level to control for possibly unobserved heterogeneity between federal states to get more accurate and robust estimates of standard errors. All analyses were adjusted for potential confounders (e.g., age, gender, education, or care experience). The conditional mean of the willingness to pay in the two-part model was calculated by multiplying the probability of having a positive willingness to pay by the expected value of the log-normal distribution, as described by Cameron and Trivedi (2005). We used the Wald test for hypothesis testing. Statistical significance was defined as *p* < 0.05 respectively *p* < 0.01, and “highly significant” as *p* < 0.001.

## Results

### Descriptive statistics

The number of respondents analyzed in the regression models was 1262, of which none had missing values in the willingness-to-pay intervals or the explanatory variables. Of these, 421 respondents each had 5 and 10 euros as the initial bid, and 420 respondents received 15 euros as their initial bid. Of the original 1350 respondents, 88 (6.5%) were identified as protest responses and thus excluded from the analysis (Havet et al., 2012). Protest responses were defined as combination of having a willingness to pay of zero and stating as reason for having no willingness to pay that financing home hospice services is either the own responsibility of individuals and everyone should pay for the services themselves or the services are public responsibility and should be financed by the government. Characteristics of the study sample, consisting of the determinants of interest and confounders in the regression models, are described in Table 1, including descriptive statistics. The representativeness check with the census data of the Austrian population showed that our sample is representative of the Austrian population regarding gender distribution, but for all other variables compared to census data, t-tests showed relevant differences in the point estimates (see Table 1). The sample characteristics are plausible, but our sample is, on average, 1.1 years younger than the reference population, and single households are underrepresented in our study, while large households with five or more persons are overrepresented. The proportion with at least high school as highest education is higher than the population average (Flatscher-Thöni et al., 2015). Net income was surveyed in income classes. Comparing the values for the 1st quartile, the median, and the 3rd quartile shows that the census values lie within the intervals of our income classes. For the income variable, 377 missing values (29.8%) were imputed.

**Table 1:** Description of the determinants used in the regression models

	Sample					Census^a^
Variable	Obs	Mean	SE	Min	Max	Mean
Age	1262	47.01	17.49	18	99	48.18^b^
Gender (female)	1262	.525	.500	0	1	.519
Education (at least high school)	1262	.479	.500	0	1	.680
Married	1262	.601	.490	0	1	.420
Single household	1262	.192	.394	0	1	.36^c^
Large household	1262	.122	.327	0	1	.18^c^
Fulltime employed	1262	.419	.494	0	1	
Parttime^d^	1262	.181	.386	0	1	
Not employed (R)^e^	1262	.399	.490	0	1	
Burgenland	1262	.032	.175	0	1	.034^f^
Carinthia	1262	.067	.251	0	1	.067^f^
Lower Austria	1262	.200	.401	0	1	.192^f^
Upper Austria	1262	.176	.381	0	1	.169^f^
Salzburg	1262	.064	.245	0	1	.063^f^
Styria	1262	.147	.355	0	1	.144^f^
Tyrol	1262	.082	.274	0	1	.084^f^
Vorarlberg	1262	.045	.208	0	1	.044^f^
Vienna (R)^g^	1262	.186	.389	0	1	.203^f^
Net income	916	2.000–2.499 euros	2.28	1	12	
Prior knowledge of hospice	1262	.674	.469	0	1	
Prior donations to support hospice	1262	.260	.439	0	1	
Hospice volunteer	1262	.010				
Use of hospice by a household member	1262	.114	.318	0	1	
Experience with death/terminal illness	1262	.825	.380	0	1	
Death of 1 close person	1262	.208	.406	0	1	
Death of 2–5 close persons	1262	.713	.452	0	1	
Death of 6–10 close persons	1262	.048	.215	0	1	
Death of more than 10 close persons (maximum n = 81)	1262	.015	.122	0	1	
Death of zero close persons (R)^h^	1262	.214	.410	0	1	
Volunteer in hospice	1262	.010	.101	0	1	
Burden to spend time with dying ones	1262	.457	.498	0	1	
Fear of death	1262	.223	.416	0	1	
Wish not to die alone	1262	.696	.460	0	1	
Care experience	1262	.199	.399	0	1	
Monthly worshipping	1262	.395	.489	0	1	
Very good health	1262	.602	.490	0	1	
Age*very good health	1262	67	25.27	0	88	
Participation at election	1262	.514	.500	0	1	
Role of God important	1262	.582	.493	0	1	
Scenario hospice as a club good	1262	.674	.469	0	1	
Initial bid 5 euros	1262	.334		0	1	
Initial bid 10 euros	1262	.334		0	1	
Initial bid 15 euros (R)^i^	1262	.333		0	1	

*Obs.* number of observations, *(R)* reference category, *SE* standard error, *WTP* willingness to pay

^a^The sample was compared with census data using t-tests

^b^The mean value for the age of the population from 18 to 100 years could only be calculated for 2021. For 2010, the data was only available in age groups

^c^According to Statistik Austria (2021)

^d^14.26% of all employed persons are part-time employees (in our sample 180 persons out of 758 employed persons) and 3.88% are marginally employed persons (in our sample, 49 persons out of 758 = 3.88%)

^e^Reference category for fulltime employed and parttime employed

^f^According to Statistik Austria (2023) (status 1 July 2010)

^g^Reference category for federal states

^h^Reference category for the number of experienced deaths of close persons

^i^Reference category for the initial bid (5, 10, or 15 euros)

The distribution of responses on the hypothetical willingness to pay across bid prices is shown in Table 2. A comparison of the proportion of response categories between the different bid levels indicates the internal validity of the responses: Our expectation that the proportion of YY(NN)-responses decreases (increases) with increasing bids is fulfilled (see Table 2). Overall, 239 people stated a willingness to pay of zero.

**Table 2:** Interval categories for the dependent variable

1st bid	2nd bid	Answer	Interval	Number of responds^a^ N (%)
15 euros	30 euros	YY	[30; ∞[	71 (5.6%)
15 euros	30 euros	YN	[15; 30[	147 (11.6%)
15 euros	7.50 euros	NY	[7.50;15[	48 (3.8%)
15 euros	7.50 euros	NN	[0;7.50[	154 (12.2%)
10 euros	20 euros	YY	[20; ∞[	109 (8.6%)
10 euros	20 euros	YN	[10;20[	139 (11%)
10 euros	5 euros	NY	[5;10[	124 (9.8%)
10 euros	5 euros	NN	[0;5[	126 (10%)
5 euros	10 euros	YY	[10; ∞[	172 (13.6%)
5 euros	10 euros	YN	[5;10[	47 (3.7%)
5 euros	2.50 euros	NY	[2.50;5[	25 (2%)
5 euros	2.50 euros	NN	[0;2.50[	100 (7.9%)
Zero WTP in follow-up question^b^				239 (18.9%)

*YY* yes, yes, *YN* yes, no, *NY* no, yes, *NN* no, no, *WTP* willingness to pay, *N* number

^a^The number also contains respondents stating a WTP of zero in the follow-up question. The proportion in parentheses refers to all 1262 respondents analyzed

^b^Only respondents who answered both bidding questions with “no” received the follow-up question if they were willing to pay at all

### Multivariate analyses

**Fig. 2:**
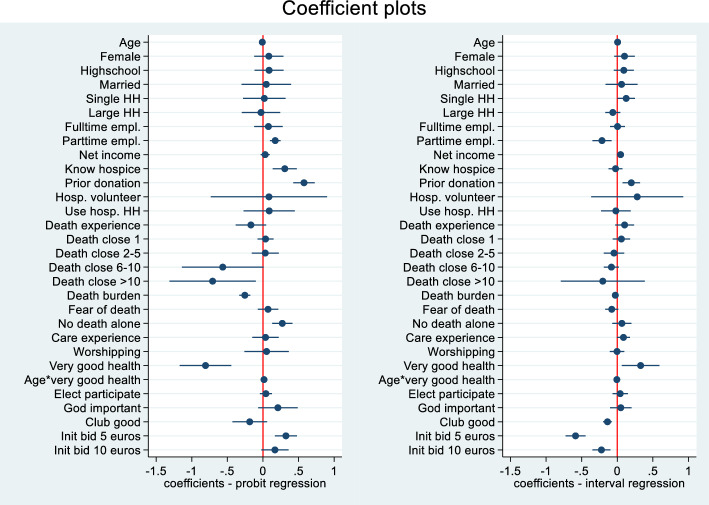
Coefficient plots for the Probit model (p(WTP > 0)) (left plot) and the interval regression model (WTP > 0) (right plot). *empl.* employed, *HH* household, *hosp.* hospice, *Init. bid* initial bid. *Notes*: Constant and state dummy variables were included in the regression model but not reported in the graphical 95% confidence intervals. Variable descriptions can be found in Table 5 in the Appendix

The Probit and interval regression analyses results are presented in Table 3 and Fig. 2. In the first stage of the two-part model the dependent variable is a dichotomous variable, where 1 indicates having a positive willingness to pay. The following determinants of interest and confounders had a statistically significant positive impact on the probability of a positive willingness to pay: *part-time employment*, *hospice knowledge*, having made *prior donations to hospice*, having the *wish not to die alone*, the interaction variable age*very good health, and a low initial bid (5 euros). Higher age, a high number of deaths of close persons (> 10), perceived *very good own health*, and the perception that spending time with dying persons is a burden significantly decreased the probability of a positive willingness to pay.

**Table 3:** Regressions results

	Regression model (dependent variable)			
	Probit model (p(wtp > 0))		Interval regression (lower wtp; upper wtp) if pwtp = 1	
main	Coefficient	95%-CI	Coefficient	95%-CI
Age	− 0.009**	− 0.015, − 0.003	0.005***	0.002, 0.008
Female	0.083	− 0.123, 0.288	0.102	− 0.044, 0.247
Highschool	0.086	− 0.118, 0.290	0.092	− 0.049, 0.233
Married	0.047	− 0.300, 0.395	0.060	− 0.165, 0.286
Single HH	0.019	− 0.280, 0.319	0.125*	0.003, 0.247
Large HH	− 0.027	− 0.297, 0.243	− 0.063	− 0.168, 0.042
Fulltime empl	0.077	− 0.123, 0.277	0.004	− 0.101, 0.109
Parttime	0.173***	0.098, 0.247	− 0.215**	− 0.349, − 0.081
Net income	0.031	− 0.033, 0.095	0.047**	0.018, 0.076
Know hospice	0.307***	0.138, 0.477	− 0.024	− 0.122, 0.074
Prior donation	0.577***	0.427, 0.728	0.197**	0.074, 0.321
Hosp. volunteer	0.085	− 0.731, 0.900	0.280	− 0.366, 0.926
Use hosp. HH	0.088	− 0.272, 0.448	− 0.020	− 0.227, 0.188
Death experience	− 0.169	− 0.383, 0.045	0.103	− 0.030, 0.236
Death of 1 close person	0.037	− 0.076, 0.150	0.058	− 0.064, 0.179
Death of 2–5 close persons	0.033	− 0.157, 0.222	− 0.046	− 0.189, 0.098
Death of 6–10 close persons	− 0.564	− 1.139, 0.010	− 0.082	− 0.188, 0.024
Death of > 10 close persons	− 0.707*	− 1.314, − 0.100	− 0.203	− 0.793, 0.387
Death burden	− 0.254***	− 0.331, − 0.177	− 0.027	− 0.072, 0.017
Fear of death	0.072	− 0.073, 0.216	− 0.078	− 0.169, 0.014
No death alone	0.272***	0.130, 0.414	0.065	− 0.069, 0.199
Care experience	0.037	− 0.149, 0.222	0.087	− 0.004, 0.178
Worshipping	0.051	− 0.261, 0.364	− 0.003	− 0.105, 0.099
Very good health	− 0.806***	− 1.169, − 0.444	0.328*	0.064, 0.592
Age*very good health	0.015***	0.007, 0.024	− 0.006*	− 0.012, − 0.000
Elect. participate	0.041	− 0.043, 0.126	0.041	− 0.067, 0.149
God important	0.210	− 0.068, 0.488	0.050	− 0.102, 0.201
Club good	− 0.186	− 0.429, 0.057	− 0.138***	− 0.199, − 0.076
Init. bid 5 euros	0.323***	0.168, 0.478	− 0.586***	− 0.726, − 0.446
Init. bid 10 euros	0.168	− 0.022, 0.359	− 0.222***	− 0.349, − 0.096
Log pseudolikelihood	− 535.179		− 1269.92	
N	1262		1023	
*Goodness of fit:*				
McFadden R^2^	n.r		0.051^a^	
Pseudo R^2^	0.126		n.r	

Constant and federal state dummies included but not reported

*CI* Confidence interval, *Init. bid* initial bid, *hosp*. hospice, *n.r*. not relevant, *pwtp* positive willingness to pay, *SE* standard error, *WTP* willingness to pay

***p < 0.001; **p < 0.01; *p < 0.05

^a^The McFadden R^2^ was calculated as: R^2^McFadden = 1-(log likelihood(crude model)/log likelihood(model with explanatory variables))

Interval regression showed a statistically significant association between the amount of willingness to pay and four sociodemographic variables (i.e., confounders), *age*, living in a *single household,* level of *net income, and part-time employment*. Older people, persons living alone, and individuals with a higher net income stated on average to have a higher level of willingness to pay. Part-time employment, which means the person responding was employed but less than 34 h, decreased the level of willingness to pay. The interaction of age with perceived health is negative, which implies that the level of willingness to pay is lower in healthy elderly than in diseased elderly.

From the determinants of interest, one variable, donations to hospice in the past three years, statistically significantly increased the level of willingness to pay.

In addition, three methodological variables, *hospice as club good, initial bid of 5 euros*, and *initial bid of 10 euros,* had a statistically highly significant negative impact on the level of willingness to pay. These methodological variables were used to account for the difference in willingness to pay between two valuation scenarios (home hospice services as publicly provided goods versus home hospice services as club good, that is, an exclusive access service) and the bid variables controlled for the different initial bids to which respondents were randomly assigned. The regression analysis reveals a statistically significant negative impact of the "club good" variable on the willingness to pay for home hospice services, which aligns with expectations. In the Austrian context, the public generally values hospice care as a public good that should be accessible to all, reflecting a societal preference for inclusivity and altruistic motivations.

### Hypothetical willingness to pay per month

The overall mean willingness to pay for a monthly contribution for home hospice services in the sample of the Austrian population was 17 euros. If the 1023 persons with a positive willingness to pay were analyzed separately, the estimated average willingness to pay increased to 20 euros. Monetary values for hypothetical willingness to pay of the determinants having a statistically significant impact on the likelihood and/or the level of a positive willingness to pay in the Probit and/or interval regression models are presented in Table 4. A review of the individual coefficients shows that willingness to pay was highest among people who had already donated to hospice services in the past three years (n = 328; 26%).

**Table 4:** Hypothetical willingness to pay per month (in 2010 euros) for specific groups of respondents

Subject area	Determinant^a^		Monetary value (euros)^b^	Difference (euros)
Prior knowledge	Prior knowledge of hospice	Yes	16.92	0.97
		No	15.95	
	Donations to hospice in prior 3 years	Yes	20.63	5.77
		No	14.86	
Experience level 2	Experienced death of more than 10 persons close to	Yes	10.51	− 6.16
		No	16.67	
Experience level 3	Spending time with dying persons is perceived as burden	Yes	15.73	− 1.53
		No	17.26	
	Wish not to die alone	Yes	17.24	2.23
		No	15.01	

^a^For determinants of interest on prior knowledge and experience that had a statistically significant effect on the probability and/or level of willingness to pay (defined as p < 0.05)

^b^Refers to the hypothetical willingness to pay per month

## Discussion

### Main findings

In our study, five determinants of willingness to pay (WTP) related to prior knowledge about (home) hospice services and experience with hospice and dying showed a statistically significant effect (three positive and two negative effects) on the probability of having a positive willingness to pay (see regression results for the Probit model (p(wtp > 0) in Table 3), but among the variables of interest only *prior donations* increased the level of willingness to pay significantly (see results of the interval regression model ((lower wtp; upper wtp) if pwtp = 1) in Table 3). The variables *prior knowledge of hospice*, *prior donations*, and the *wish not to die alone* significantly increase the probability of having a positive willingness to pay while experienced death of more than 10 close persons (maximum n = 81) during the last five years and perceiving it as a burden to spend time with dying ones lead to a significantly lower probability of having a positive willingness to pay (Probit model (p(wtp > 0)) in Table 3).

Besides *prior donations,* the interval regression analysis revealed a statistically significant positive impact of higher age, higher net income, living in a single household, and perceived very good health, and a negative effect for working parttime, hospice as club good, and the two variables for the starting bids, five euros and ten euros. By focusing on the influence of the respondents' prior knowledge of home hospice services and experience with death and dying in the average population, we show how awareness of and experience with available home hospice services can increase utility. Thus, society's benefit from hospice could be increased by creating awareness about existing services, thereby increasing access to previously uninformed persons. Investments in the maintenance and expansion of home hospice services and communication of the benefit of these services to the public, therefore, have a welfare-enhancing potential.

### Context to literature

Research on the monetary valuation of end-of-life health services beyond medical treatment remains limited. There are no studies available that allow a direct comparison of our results of the economic evaluation of home hospice services. However, a few studies have evaluated society's willingness to pay for healthcare services that bear at least some resemblance to hospice services but a detailed comparison of results is beyond the scope of this paper.

One study evaluated the willingness to pay for a suicide prevention intervention in Japan (Sueki, 2017). The willingness to pay for the intervention was assessed from a social perspective, depending on the respondent's attitude, hospice knowledge, and prior experience. Similar to our results, respondents showed a significantly higher willingness to pay among individuals who have previous information about the potential effects or benefits of the intervention. A second study evaluated the willingness to pay for hospital-based hospice services in Korea, finding a positive income elasticity in willingness to pay for hospice (Kim et al., 2011). However, in the latter study, the contingent valuation focused on the care of cancer patients (Kim et al., 2011).

Four studies with discrete choice experiments showed that people may also have a willingness to pay to avoid a stay in a nursing facility (also called institutionalization aversion), especially in long-term care. Three studies from the Netherlands, Denmark, and Germany examined the value of home-based long-term care, highlighting a high perceived value for services addressing social needs (Amilon et al., 2020; Lehnert et al., 2018; Nieboer et al., 2010). Findings from these studies suggest that quality and familiarity with care providers significantly influence willingness to pay, a conclusion that is also relevant for home hospice care. The fourth study by (Costa-Font, 2017), put the main focus on individuals' willingness to pay to avoid institutionalization in relationship to a potential impairment and socioeconomic characteristics. However, with respect to the home hospice services we evaluated in our study, avoiding unplanned hospitalizations is a goal, but the value of home hospice services is not primarily in avoiding institutionalization because the mobile teams provide services where the patient is, whether at home, in a nursing home, or elsewhere (see also Online Resource ESM 1 for more details) (Pelttari et al., 2021).

Regarding economic aspects, studies have examined the cost-saving potential of hospice care, but primarily focusing on medical treatment which is not part of our home hospice services (Huang et al., 2020). A study in Hungary found a strong correlation between previous donations and actual willingness to pay for informal payments for health services (Baji et al., 2014) which was also the case in our study, as previous donations to hospices have an impact on willingness to pay. The results of a study from the United States commissioned by the National Association for Home Care & Hospice (NAHC) and the National Hospice and Palliative Care Organization (NHPCO) underline the value of hospice for patients and their families (NORC at the University of Chicago, 2023). However, in the United States the medical treatment is main part of the hospice service assessed and the findings are not directly comparable to our findings but the study also confirmed findings of prior studies that hospice care adds value to patients, family members, and caregivers by increasing satisfaction, improving pain control, and enhancing both patients’ quality of life but also of their families and caregivers by reducing physical and emotional distress (NORC at the University of Chicago, 2023).

Although social support is a key element of hospice care, it is rarely quantified in economic terms. Our study, therefore, evaluated the hypothetical WTP for home hospice services in Austria, where care is largely provided by trained volunteers (see ESM 1 for details).

### Strengths and limitations

A strength of our study is that we relied in the design of our empirical model on previous empirical evidence retrieved in a systematic review (Steigenberger et al., 2022) to identify relationships between determinants and willingness to pay. We put effort into controlling potential biases. To avoid anchoring bias, we used three different initial bids with levels based on the range of responses from a pre-test and added open-ended questions, if respondents answered twice “no” to distinguish zero from positive WTP values. As described by Rotteveel et al. (2020) and Havet et al. (2012), we handled the influence of respondents with a willingness to pay of zero by excluding protest answers (Havet et al., 2012) from the analysis and using a two-step regression analysis with a Probit model on positive willingness to pay in the first step and an interval regression with respondents having a positive willingness to pay in a second step. Our results are robust concerning the type of regression model (bivariate vs. two-part model) and the number of WTP responses used (single-bounded vs. double-bounded). As Carson and Hanemann (2005) discuss, respondents' answer to the second question in the double-bounded dichotomous choice format does not necessarily refer to the same good/service as in the initial question. This possible difference in perception regarding the good/service to be evaluated is caused by the changing bid between the first and second question. Furthermore, anchoring and “yea-saying” effects could influence respondents' answers, especially in sequential WTP questions. In a robustness check, we therefore examined whether incentive incompatibility and anchoring affect our results by using only the respondents' answer to the first WTP question. This specification produced similar results in terms of the influence of explanatory variables on WTP. Thus, we believe that neither incentive incompatibility nor anchoring strongly biased our results.

As with all stated preference studies, our study faces the common risk of hypothetical bias (Andor et al., 2017; Johnston et al., 2017). Hypothetical bias describes the phenomenon that the evaluation in a hypothetical scenario may be different from the evaluation in a real-world setting. In addition, loss aversion could be another reason for an upward bias in the evaluation because the contingent valuation question is formulated as payment to prevent the loss of home hospice services. Loss aversion refers to the tendency to value losses higher than gains (Tversky & Kahneman, 1981). Both hypothetical bias and loss aversion could result in a higher stated than an actual willingness to pay. However, our results comply with some validity checks (decreasing marginal utility of income and an increase of no-answers with increase of costs) and provide evidence that the identified willingness to pay is more than just an endorsement of home hospice services but can be interpreted as an economic utility measure, whose level informs about the strength of preferences (Carson & Hanemann, 2005). It may also be seen as a limitation that we define home hospice services relatively broadly. Therefore, we cannot exclude with certainty that, in addition to psychosocial support and bereavement care, which are primarily provided by home hospice teams, palliative care services may also have been assessed by some respondents. The issue that medical services were also assessed by the respondents and that it was not entirely clear what home hospice services are was also present in a systematic review. Plagg et al. differentiated between palliative care, hospice care and end-of-life care, but due to an unclear definition of the concepts, the services had to be summarized under the collective term hospice and palliative care, which was stated as a limitation of the systematic review (Plagg et al., 2023).

A further limitation of our research is the time of data collection. The presented empirical evidence is based on data collected in Austria in 2010, when home hospice care was already systematically anchored in the Austrian healthcare system. Home hospice services have not been subject to major legal changes regarding the development and their provision ever since. We acknowledge that this represents a significant limitation, particularly considering changing societal attitudes and perceptions toward home hospice services, especially following the COVID-19 pandemic.

It is possible that the COVID-19 pandemic may have influenced the perception of and willingness to pay for home hospice services among the Austrian population, although the direction and extent of this impact is unclear. While it is likely that the pandemic has affected people’s attitudes toward mortality risks, as discussed in the literature (e.g., Hammitt, 2020), our empirical investigation focuses on a specific type of healthcare service at the end of life that does not pertain to any particular illness or mortality risk.

Furthermore, literature documents various challenges faced by palliative care and hospice services during the pandemic, such as issues with availability, isolation, barriers to communication, and disruptions in systems (Bone et al., 2020; Etkind et al., 2020; Gergerich et al., 2021; Hammitt, 2020). Nevertheless, these studies do not specifically address changes in societal valuation of these services. To date, no research has been published on the impact of the pandemic on social services at the end of life in society from the demand side. However, two publications have addressed the supply side, that is, a report on the impact of the COVID-19 pandemic on the social services sector commissioned by the Federation of European Social Employers and the European Federation of Public Service Unions (FORESEE, 2022) recommending investments in the social services sector also in pandemic preparedness plans and a systematic review (Plagg et al., 2023) assessing challenges for providers of hospice and palliative care during disasters, as the COVID-19 pandemic. The systematic review by Plagg et al. (2023) highlighted the potential for hospice care to alleviate the psychological distress experienced by healthcare workers, for instance, by assisting in the clarification of advance directives and the minimization of unwanted resuscitation measures, which could be interpreted as increased valuation of home hospice services by providers but there is no assessment of the demand side. Therefore, it may be, that either the baseline (intercept) WTP increased after the pandemic or there are simply more people having experienced death in their families and social environments.

However, it remains unclear whether the old "normal" referring to the time prior to the pandemic is actually so different from the new "normal" post pandemic. As stated by (Tully & Meyvis, 2017) in their work on forgetting to remember experiences, it might also be that experiences during the pandemic did have a mild effect on the current situation in the Austrian society.

Hence, we strongly believe our data provide informational value for non-pandemic times to guide resource allocation decisions. Further research should investigate this aspect in more detail and include pandemic experiences into the set of variables.

### Policy implications

We have measured the societal benefits resulting from home hospice services in monetary terms. Such a monetary valuation of home hospice services was not available before. Besides costs, the monetary evaluation of benefits is a pre-condition to conduct a cost–benefit analysis for efficient resource allocation for home hospice services. We recommend improving the communication and information regarding home hospice services, for example, in doctors' practices or via social media, since one-third of the sample (33%) did not know (home) hospice services and might not be able to access them if needed. It is in the nature of home hospice services that people realize its value primarily when a need arises and people get affected directly or by experiencing social needs at the end of life, for example, in close persons.

Our findings may also be relevant to funding discussions in other countries since this study is the first evaluation of home hospice services based on statements of (potential) beneficiaries focusing on what determines the societal valuation of home hospice services in a general setting (i.e., not related to a specific illness). We determined the contribution of knowledge and experience to the monetary value of home hospice services for society. There is also potential for (volunteer) home hospice teams to reduce the pressure on the healthcare system, both during times of crisis and normal times. Assessments conducted by the Welsh National Health Service have demonstrated that the formation of new partnerships between professionals and volunteers have the potential to enhance health and well-being of patients, caregivers, healthcare professionals, and other pertinent stakeholder groups. Furthermore, these partnerships have the capacity to generate a social return on investment (Liddell, 2022).

## Conclusions

Home hospice services have value for society: our cross-sectional willingness to pay study suggests that a person's prior knowledge of hospice and experience with death and dying increases the probability of having a positive willingness to pay for home hospice services. For persons who donated previously to hospices, the stated level of willingness to pay increased. Our analysis could not show a statistically significant effect for further variables. We were able to provide concrete numbers for the value of home hospice services. Ensuring access to home-based hospice services for all patients and caregivers, when needed, is an important issue to enable aging in dignity and, thus, dying in dignity. Evidence to justify societal expenditure still needs to be improved; therefore, our research contributes to recent policy discussions.

## Electronic supplementary material

Supplementary file 1

Supplementary file 2

## Data Availability

The dataset analyzed during the current study and the STATA code will not be publicly available since further analyses will be conducted and published but it can be requested from the corresponding author on reasonable request.

## Appendix

See Table 5.

**Table 5:** Explanatory variables

Variable (alphabetic)	Short form of variable name	Description	References for the link on WTP in addition to determinants from a systematic review^a^
		*Sociodemographic Variables*	
Age	Age	Age of PR	Hansen et al. (2013), Read and Read (2001)
Gender (female)	Female	Dummy. Equals 1 if PR is female	
Education (high school)	Highschool	Dummy. Equals 1 if PR has at least a high school diploma (Matura in Austria)	
Married	Married	Dummy. Equals 1 if PR is married or lives in a living community	
Single household	Single HH	Dummy. Equals 1 if PR lives in a single household	
Large household	Large HH	Dummy. Equals 1 if the household is 5 persons or bigger	
Fulltime employed	Fulltime empl	Dummy. Equals 1 if PR works full-time (> 34 h/week)	
Part-time employed	Parttime	Dummy. Equals 1 if PR is employed part-time (PR stated to be employed but < 34 h/week)	
Federal state^b^	Federal state	9 dummies, one for each federal state of Austria: 1: "Burgenland", 2: "Carinthia", 3: "Lower Austria", 4: "Upper Austria", 5: "Salzburg", 6: "Styria", 7: "Tyrol", 8: "Vorarlberg", 9: "Vienna"	
Net income	Net income	Personal monthly net income. The variable has 12 attribute groups plus “no answer”	Varian (2010) (pp. 65f and pp. 116f)
		*Prior knowledge*	
Prior knowledge of hospice	Know hospice	Dummy. Equals 1 if PR has knowledge about hospice	Kim et al. (2011)
Prior donations to support hospice	Prior donation	Dummy. Equals 1 if PR donated to hospice during the last 3 years	
Volunteer in hospice	Hosp. volunteer	Dummy. Equals 1 if PR is volunteering in hospice care	
		**Experience** level 1	
Use of hospice by a household member	Use hosp. HH	Dummy. Equals 1 if PR states hospice has been used by someone in their household	Sana et al. (2020): a positive impact of awareness on WTP
		**Experience** level 2	
Experience with death/terminal illness	Death experience	Dummy. Equals 1 if PR has experience with death (personal, job, volunteer)	Bouvy et al. (2011): affected patients would pay more
Death of 1 close person	Death close 1	Dummy. Equals 1 if PR experienced the death of 1 close person during the last 5 years	Hong et al. (2018): state the role of death experiences
Death of 2–5 close persons	Death close 2–5	Dummy. Equals 1 if PR experienced the death of 2–5 close persons during the last 5 years	
Death of 6–10 close persons	Death close 6–10	Dummy. Equals 1 if PR experienced the death of 6–10 close persons during the last 5 years	
Death of more than 10 close persons	Death close > 10	Dummy. Equals 1 if PR experienced the death of more than 10 close persons during the last 5 years	
		**Experience** level 3	
Burden to spend time with dying ones	Death burden	Dummy. Equals 1 if PR states spending time with dying persons is a burden	
Fear of death	Fear of death	Dummy. Equals 1 if PR states the expectation of death is scary	McClain et al. (2003): impact of spiritual well-being and end-of-life despair on WTP; Weber et al. (2012): fear functions as motivation to reduce perceived risk and take action
Wish not to die alone	No death alone	Dummy. Equals 1 if PR states that not dying alone is important	Chochinov et al. (2011) Statements on death and dying
**Other variables**			
Care experience	Care experience	Dummy. Equals 1 if PR is a caregiver	Urwin et al. (2021), Voltaire (2015)
Monthly worshipping	Worshipping	Dummy. Equals 1 if PR goes worshipping at least once per month	Neustadt (2011)
Very good health	Very good health	Dummy. Equals 1 if PR reported having excellent or very good health	Hutjens (2014)
Age* very good health	Age*very good health	Interaction term of age times perceived very good health	
Participation at election	Elect. participate	Dummy. Equals 1 if PR has a preferred party	Adler and Goggin (2005): Proxy for civil engagement
Role of God important	God important	Dummy. Equals 1 if the role of God is seen as necessary by PR	World Value Survey (V185–187 questions on religiosity) (World Value Association, 2009)
Hospice as club good (scenario)	Club good	Dummy for the variant. Equals 1 if PR is in the scenario with hospice defined as a club good, i.e., an exclusive access service; else, hospice is defined as a public good	Gyrd-Hansen (2018), Azar et al. (2018)
Initial bid 5 euros	Init. bid 5 euros	Dummy. Equals 1 if the initial bid was 5 euros	
Initial bid 10 euros	Init. bid 10 euros	Dummy. Equals 1 if the initial bid was 10 euros	

*HH* household, *Init. bid* initial bid, *hosp*. hospice, *PR* person responding to the question, *WTP* willingness to pay

^a^All explanatory variables relate thematically to determinants identified in a systematic review by Steigenberger et al. (2022)

^b^For each federal state, a dummy variable was created
