# Supplementary material for: Influence of prior knowledge and experience on willingness to pay for home hospice services: a contingent valuation study
Source: Int J Health Econ Manag. 2025 Mar 25;25(3):293–315. doi: 10.1007/s10754-025-09393-8 (PMC12568916; doi:10.1007/s10754-025-09393-8)
Supplement: Supplementary file 1 [file 10754_2025_9393_MOESM1_ESM.pdf]

Content refers to: Steigenberger, C., Leiter, A.M., Siebert, U., Schusterschitz, C., Flatscher-Thoeni, M. (2025). Influence of prior knowledge and experience on willingness to pay for home hospice services: a contingent valuation study. *International Journal of Health Economics and Management.* DOI: 10.1007/s10754-025-09393-8
Email address corresponding author: caroline.steigenberger@umit-tirol.at

# Electronic Supplementary Material 1: Hospice in Austria

1. Home hospice care in Austria ... 1
2. Providers of home hospice services ... 1
3. Scope of home hospice services ... 2
4. Target group of home hospice services ... 2
5. Figures related to home hospice services in Austria ... 2
6. Costs and funding of home hospice service provision ... 2
7. Outlook: current developments in Austria ... 3
References ... 3

## 1. Home hospice care in Austria

Home hospice teams support caregivers and patients and aim to meet the emotional and social needs of patients and their relatives. They support families in daily activities, with symptom management, and provide bereavement support at the end of life.[1] A home hospice team comprises qualified volunteers and at least one permanent coordinator. Palliative patients and their families receive social and spiritual support, both during the illness and in the mourning phase. In addition, home hospice teams work closely with other healthcare providers, such as specialized palliative care services.[2]

Home hospice services are supplementary services for affected persons in addition to palliative care. Palliative care is provided by healthcare professionals.

There is no legal basis for the involvement of volunteers in hospice and palliative care in Austria, but there are recommendations from the independent national umbrella organization Hospice Austria and the Austrian Ministry of Health. A more detailed description of the development of home hospice services in Austria and the involvement of volunteers can be found in a book chapter on "Volunteering in hospice and palliative care in Austria".[3]

## 2. Providers of home hospice services

In Austria, the provision of home hospice services is sustainably integrated into the health care system. Home hospice services are coordinated and organized by members of the umbrella organization *Hospice Austria*, the independent Austrian National Association for Hospice and Palliative Medicine. It was founded in 1993 and has since been promoting and improving the availability and quality of hospice and palliative care in Austria. *Hospice Austria* is responsible for the coordination and organization of home hospice services.

Full members of the umbrella organization are regional hospice and palliative care associations, health institutions, hospice initiatives, and educational institutions from the hospice and palliative sector in all federal states. There are also four supra-regional members: *Caritas Austria*, the *Austrian Red Cross*, *Diakonie Austria*, and the *Vinzenz Group*.[4] Full members also take responsibility for the implementation, education, and training of home hospice teams.

A complete list of individual members is available on the website of *Hospice Austria*, grouped by federal state.[5] The division of the organization into federal state associations reflects the decentral structure of the Austrian health care system.

Email address corresponding author: caroline.steigenberger@umit-tirol.at

## 3. Scope of home hospice services

Home hospice teams in Austria provide services supplementary to palliative medical care to support patients and their families during the dying process, including pain and symptom management, emotional and spiritual support, assistance with daily activities, and medication management.[2] Mobile teams provide many services at the place where the patient is. They reduce the level of distress for patients (and family members) and can help to avoid stressful admissions to the hospital due to fewer serious medical complications. In the following, we describe home hospice services related to these areas in more detail. Home hospice teams are trained to manage pain and other symptoms associated with advanced illness. They work closely with the patient's attending physician to ensure the patient is as comfortable as possible.

The area of emotional and spiritual support for both the patient and family members is very comprehensive and may include counseling, spiritual guidance, and support groups, such as bereavement groups. Bereavement groups are primarily for family members and loved ones of patients who have received care and died, but beyond that, the groups are usually open to all.

Assistance with daily activities is also a main part of the service at the patient's home, enabling them to stay home during the last phase of their life. Hospice teams help patients with daily activities such as bathing, dressing, and eating. They can also help with housework, shopping, and meal preparation. Home hospice teams can also help patients manage their medications, including administering medicines and coordinating refills.

Particularly in the case of oncologic malignancies, patients typically live with the disease for several months to years before death, and the end-of-life phase can extend over months.[6] Without home hospice services, adequate medical care is often impossible on-site, and patients are administered to the hospital.

## 4. Target group of home hospice services

The target groups of home hospice services are all palliative patients, their relatives and friends, and mourners. As an access criterion, it is sufficient that the palliative patient and their relatives or mourners wish to be accompanied.[7]

## 5. Figures related to home hospice services in Austria

This section provides some facts and figures related to home hospice services in Austria. In 2020, there were 173 home hospice teams for adults that cared for a total of 9,898 patients in Austria. Besides patients, there are approximately 3-5 relatives per patient who receive bereavement support (provided to 1730 mourners in 2020).[2]

Despite the wish to die at home, as described in the manuscript, the actual place of death for 49.06% was in the hospital and 18.56% in another institution (e.g., nursing or retirement home).[8] Still, 26,38% died at home, which might not have been possible without home hospice teams. The places of service provision of home hospice teams were primarily at home (33.0%), in the nursing home (21.4%), and in palliative care wards (27.9%).[2]

For more information on the geographic and political context of Austria and socio-demographic indicators, we refer to the health system review on Austria published by the European Observatory on Health Systems and Policies on behalf of the World Health Organization.[9]

## 6. Costs and funding of home hospice service provision

For the provision of home hospice services, costs incur for permanent coordinators, trainers for hospice companions, travel costs, infrastructure such as buildings, and other costs directly or indirectly related to home hospice services. In Austria, these costs are covered by public subsidies, donations from the

population, and volunteers donating their time for the provision of home hospice services. The importance of reliable public funding increased with the rising number of hospice teams.

There are no costs for the patient or their families to receive home hospice services.

The consequences of a loss of public funding would be that the infrastructure can no longer be maintained, which ensures that home hospice services can be provided in high quality by trained persons, which is highly important for persons in contact with the vulnerable group of dying persons.

## 7. Outlook: current developments in Austria

A parliamentary Enquette Commission on Dignity at the End of Life[10] was established based on a clear political commitment to enable aging in dignity and thus dying in dignity. A nationwide expansion of hospice care was implemented in the current Austrian government program 2020-2024[11] as national priority. The relevance of meeting psychosocial needs at the end of life has also been emphasized by a recent decision of the Austrian Federal Constitutional Court related to assisted suicide and self-determined dying in 2020.[12]

According to the umbrella organization *Hospice Austria*, the development of standard financing is essential because there are very different financing models of specialized hospice and palliative care facilities throughout Austria, which have grown independently of each other in the individual federal states. Regular funding would be important to safeguard existing services and further expansion in line with demand.[2] Meanwhile, the Austrian National Public Health Institute has elaborated an evaluation on the feasibility of regular public funding, which is currently being worked out at the political level, but the results are only available in a summarized form.[13]

In May 2022, a broad majority of the Austrian parliament approved a law referring to the regular funding of hospice and palliative care and endowed a public fund with 108 million euros.[14]

Evidence to justify societal expenditure still needs to be improved; thus, our research contributes to recent policy discussions.

## References

1. de Graaf E, Zweers D, Valkenburg A, et al. Hospice assist at home: does the integration of hospice care in primary healthcare support patients to die in their preferred location - A retrospective cross-sectional evaluation study. Palliat Med 2016; 30: 580-586. 2016/01/28. DOI: 10.1177/0269216315626353.
2. Pelttari L, H. PA, Nemeth C, et al. Hospice and palliative care in Austria 2020. Data report of specialized hospice and palliative care facilities, educational work, and projects in primary care. [Hospiz- und Palliative Care in Österreich 2020. Datenbericht der spezialisierten Hospiz- und Palliativeinrichtungen, der Bildungsarbeit sowie der Projekte in der Grundversorgung.], 2021.
3. Pelttari L and Pissarek AH. Chapter 4: Volunteering in hospice and palliative care in Austria. In: Scott R and Howlett S (eds) The Changing Face of Volunteering in Hospice and Palliative Care. OUP Oxford, 2018.
4. Hospice Austria. Organizational structure [Organisationsstruktur], https://www.hospiz.at/organisationsstruktur/ (accessed: 8 February 2023), 2023.
5. Hospice Austria. Overview of institutions. Hospice teams. [Einrichtungsübersicht. Hospizteams.], https://www.hospiz.at/einrichtungsuebersicht/ (accessed: 8 February 2023), 2023.
6. Radbruch L, Payne S and null. Standards und Richtlinien für Hospiz- und Palliativversorgung in Europa: Teil 2. Weißbuch zu Empfehlungen der Europäischen Gesellschaft für Palliative Care (EAPC), 2011; 12: 260-270. DOI: 10.1055/s-0031-1276957.
7. Austrian National Public Health Institute. Abgestufte Hospiz- und Palliativversorgung für Erwachsene. 2014. Wien, Austria: Bundesminesterium für Gesundheit.

8. KAGes. Places of death in Austria - adults, https://www.hospiz.at/wordpress/wp-content/uploads/2021/02/Sterbeorte_Alle_18_2019.pdf (accessed: 8 February 2023), 2021.
9. Bachner F, Bobek J, Habimana K, et al. Austria: Health system review. Health Systems in Transition. 2018.
10. Mückstein E. Report of the Parliamentary Enquete Commission on Dignity at the End of Life. 2015.
11. The Republic of Austria. Care. Out of responsibility for Austria Government Program 2020-2024 [Aus Verantwortung für Österreich Regierungsprogramm 2020–2024]. 2020, pp. 172-176.
12. Austrian Constitutional Court. It is unconstitutional to prohibit any form of assisted suicide without exception, https://www.vfgh.gv.at/medien/Toetung_auf_Verlangen_Mithilfe_am_Suizid.php (accessed: 16 May 2022), 2020.
13. Austrian National Public Health Institute. Regular funding in hospice and palliative care for adults, children, adolescents and young adults. Analysis of the financing models of all offers of specialized hospice and palliative care in Austria. Publicly provided summary [Regelfinanzierung in der Hospiz- und Palliativversorgung für Erwachsene, Kinder, Jugendliche und junge Erwachsene. Analyse der Finanzierungsmodelle aller Angebote der spezialisierten Hospiz- und Palliativversorgung in Österreich. Öffentlich bereitgestellte Kurzfassung]. 2021.
14. Austrian Parliament. National Council: Broad majority in favor of regular funding for hospice and palliative care. Hospice and palliative care fund endowed with € 108 million, https://www.parlament.gv.at/PAKT/PR/JAHR_2022/PK0173/index.shtml# (accessed: 19 June 2022), 2022.
