# Supplementary material for: Influence of prior knowledge and experience on willingness to pay for home hospice services: a contingent valuation study
Source: Int J Health Econ Manag. 2025 Mar 25;25(3):293–315. doi: 10.1007/s10754-025-09393-8 (PMC12568916; doi:10.1007/s10754-025-09393-8)
Supplement: Supplementary file 2 [file 10754_2025_9393_MOESM2_ESM.pdf]

Content refers to: Steigenberger, C., Leiter, A.M., Siebert, U., Schusterschitz, C., Flatscher-Thoeni, M. (2025). Influence of prior knowledge and experience on willingness to pay for home hospice services: a contingent valuation study. *International Journal of Health Economics and Management.* DOI: 10.1007/s10754-025-09393-8
Requests related to this questionnaire can be sent to andrea.leiter@uibk.ac.at.

Online supplementary 2: Questionnaire (original version is in German)

Survey number: A-14653
Subject: Hospice services in Austria
Target group: n=1,000 Representative for population aged 18 and over

---

Where personal expressions are used in this questionnaire, these include women and men alike.

Hello, my name is... I am from the SPECTRA market research institute. We are conducting a representative survey on volunteering and hospice on behalf of the University of Innsbruck.

Could I please speak to the person in your household who is 18 years old and whose birthday is next or the youngest person who is over 18 years old?

Would you be so kind as to take part in a 25-minute survey? Your personal opinion is required, so please answer the questions as openly and honestly as possible. Your data and answers will, of course, be treated strictly confidentially and anonymously.

S1.
Gender:
1: Male
2: Female

S2.
May I ask you about your exact age?

1.
Do you work in non-profit organizations or associations such as the Red Cross, social welfare institutions, sports clubs, etc. on a voluntary basis, i.e., without pay?

DO NOT READ!
1: Yes → **Continue to question 2**
2: No → **Continue to question 3**
3: Not specified → **Continue to question 3**

The original validated version of this questionnaire is in German. This translation into English was created with the translation tool of the European Commission eTranslation version 13.4 (https://webgate.ec.europa.eu/etranslation/about.html) and is not validated.
All rights are reserved to the authors in accordance with applicable copyright and intellectual property laws. Any unauthorized use or reproduction of this questionnaire will result in legal action.

Content refers to: Steigenberger, C., Leiter, A.M., Siebert, U., Schusterschitz, C., Flatscher-Thoeni, M. (2025). Influence of prior knowledge and experience on willingness to pay for home hospice services: a contingent valuation study. *International Journal of Health Economics and Management.* DOI: 10.1007/s10754-025-09393-8
Requests related to this questionnaire can be sent to andrea.leiter@uibk.ac.at.

2.
There is a number of reasons why people can volunteer. For each statement, please tell me if this is a reason why **you** are volunteering.

DO NOT READ!
1: Yes
2: No
3: Don't know
4: Not specified
READ!
a) Because it makes me part of a nice community
b) Because the activity gives me the opportunity to get to know myself better
c) Because it is important to me to support people in need
d) Because it makes me feel recognized and valued
e) Because my family/friends are volunteers
f) Because the activity represents a meaningful balance to my professional activity
g) Because the activity gives me satisfaction that I do not find in my profession
h) Because the activity gives me satisfaction that I do not find in my family
i) Because it improves my professional opportunities
j) Because I feel the need to contribute to the change of our society
k) Because I myself was in a similar situation to people for whom I am now volunteering
l) Because I enjoy this activity

→Continue to question 4

3.
There are a number of reasons why people do not volunteer. For each statement, please tell me whether this is a reason why volunteering is not an option for you at the moment.

DO NOT READ!
1: Yes
2: No
3: Don't know
4: Not specified

READ!
a) Have too little free time available
b) Have inflexible working hours (shift work and the like)
c) Have other interests

The original validated version of this questionnaire is in German. This translation into English was created with the translation tool of the European Commission eTranslation version 13.4 (https://webgate.ec.europa.eu/etranslation/about.html) and is not validated.
All rights are reserved to the authors in accordance with applicable copyright and intellectual property laws. Any unauthorized use or reproduction of this questionnaire will result in legal action.

Content refers to: Steigenberger, C., Leiter, A.M., Siebert, U., Schusterschitz, C., Flatscher-Thoeni, M. (2025). Influence of prior knowledge and experience on willingness to pay for home hospice services: a contingent valuation study. *International Journal of Health Economics and Management.* DOI: 10.1007/s10754-025-09393-8
Requests related to this questionnaire can be sent to andrea.leiter@uibk.ac.at.

d) The question has never arisen for me
e) Would like to use my free time differently
f) Does not wish to make a permanent commitment
g) I have never been asked to volunteer
h) Physical requirements are too high
i) Mental health requirements are too high
j) Cannot identify me with the group of volunteers
k) No or too little remuneration

3a.
In general, can you imagine working in a non-profit organization if you would receive a remuneration for it?

DO NOT READ!
1: Yes
2: No
3: Don't know
4: Not specified

4.
Does your partner work on a voluntary basis?

DO NOT READ!
1: Yes
2: No
3: I don't have a partner
4: Don't know
5: Not specified

5.
Do friends or relatives of yours work on a voluntary basis?

DO NOT READ!
1: Yes
2: No
3: Don't know
4: Not specified

The original validated version of this questionnaire is in German. This translation into English was created with the translation tool of the European Commission eTranslation version 13.4 (https://webgate.ec.europa.eu/etranslation/about.html) and is not validated.
All rights are reserved to the authors in accordance with applicable copyright and intellectual property laws. Any unauthorized use or reproduction of this questionnaire will result in legal action.

Content refers to: Steigenberger, C., Leiter, A.M., Siebert, U., Schusterschitz, C., Flatscher-Thoeni, M. (2025). Influence of prior knowledge and experience on willingness to pay for home hospice services: a contingent valuation study. *International Journal of Health Economics and Management.* DOI: 10.1007/s10754-025-09393-8
Requests related to this questionnaire can be sent to andrea.leiter@uibk.ac.at.

6.
Apart from contributions to religious communities, do you support non-profit organizations financially, e.g., in the form of membership fees or donations?

DO NOT READ!
1: Yes
2: No
3: Not specified

7.
There is a number of reasons why people can volunteer. Please tell me if this could be a reason for **other people** to volunteer.

DO NOT READ!
1: Yes
2: No
3: Don't know
4: Not specified

READ!
a) Because it makes you part of a nice community
b) Because such an activity offers the opportunity to get to know oneself better
c) Because it is important for people to support those in need
d) Because it makes you feel recognized and valued
e) Because the family/friends are volunteers
f) Because the activity represents a reasonable compensation for the professional activity
g) Because one fulfills the activity with satisfaction, which one cannot find in the profession
h) Because one fulfills the activity with satisfaction that one cannot find in the family
i) Because it improves professional opportunities
j) Because one has the need to contribute to the change of society
k) Because you yourself were in a similar situation as people for whom you are now volunteering
l) Because one feels joy in this activity

8.
Now let's talk about hospice facilities. Do you know hospice facilities, e.g., palliative care units, or mobile home hospice teams?

DO NOT READ!
1: Yes

The original validated version of this questionnaire is in German. This translation into English was created with the translation tool of the European Commission eTranslation version 13.4 (https://webgate.ec.europa.eu/etranslation/about.html) and is not validated.
All rights are reserved to the authors in accordance with applicable copyright and intellectual property laws. Any unauthorized use or reproduction of this questionnaire will result in legal action.

Content refers to: Steigenberger, C., Leiter, A.M., Siebert, U., Schusterschitz, C., Flatscher-Thoeni, M. (2025). Influence of prior knowledge and experience on willingness to pay for home hospice services: a contingent valuation study. *International Journal of Health Economics and Management.* DOI: 10.1007/s10754-025-09393-8
Requests related to this questionnaire can be sent to andrea.leiter@uibk.ac.at.

2: No
3: Don't know
4: Not specified

9.
IF CODE 1 (YES) IN QUESTION 8: Then you know that hospice facilities care for seriously ill, dying people and their relatives either in facilities such as hospitals or through mobile home hospice teams, directly in the households.

IF CODE 2-4 IN QUESTION 8: Hospice facilities care for seriously ill, dying people and their relatives either in facilities such as hospitals or through mobile home hospice teams, directly in the households.

TO ALL: Activities include grief counseling, death counseling, organization/execution of day-to-day errands, conducting conversations, etc.

Is there a hospice facility in your place of residence?

DO NOT READ!
1: Yes
2: No
3: Don't know

---

10. ***DO NOT REQUEST IF ANSWERED TO QUESTION 1 CODE 2 (NO)!***
Are you a volunteer in a hospice facility or a mobile home hospice team?

DO NOT READ!
1: Yes
2: No
3: Not specified

11.
Has your household ever used hospice services, whether home hospice services at home or in facilities such as hospitals?

DO NOT READ!
1: Yes
2: No
3: Don't know
4: Not specified

The original validated version of this questionnaire is in German. This translation into English was created with the translation tool of the European Commission eTranslation version 13.4 (https://webgate.ec.europa.eu/etranslation/about.html) and is not validated.
All rights are reserved to the authors in accordance with applicable copyright and intellectual property laws. Any unauthorized use or reproduction of this questionnaire will result in legal action.

Content refers to: Steigenberger, C., Leiter, A.M., Siebert, U., Schusterschitz, C., Flatscher-Thoeni, M. (2025). Influence of prior knowledge and experience on willingness to pay for home hospice services: a contingent valuation study. *International Journal of Health Economics and Management.* DOI: 10.1007/s10754-025-09393-8
Requests related to this questionnaire can be sent to andrea.leiter@uibk.ac.at.

12.
Have you or one of your household members supported hospice facilities financially in the last 3 years, e.g., in the form of donations?

DO NOT READ!
1: Yes
2: No
3: Don't know
4: Not specified

13.
Now let's talk more about mobile home hospice teams. Mobile home hospice teams offer assistance such as death and mourning support directly in the household of the person cared for. In 2008, around 2,800 people spent more than 300,000 hours in mobile home hospice teams throughout Austria. These home hospice services have so far been provided through public funds.

VARIANT 1 – CLUB GOOD (n=700 interviews)
Assume that the public funds are no longer available and that funding is to be provided by membership fees in the future, i.e., any person can voluntarily become a member of the hospice and, if active, use the services of mobile home hospice teams at no additional cost. Non-members have to look for a different solution for needed assistance.

VARIANT 2 – PUBLIC GOOD (n=300Interviews)
Assume that public funds are no longer available and that funding is to be provided through earmarked contributions in the future, i.e., any person receiving income above the subsistence level must make a contribution and can claim the services of mobile home hospice teams in return. For humanitarian reasons, people who do not pay contributions can also use the services of mobile home hospice teams.

Electronic Data Processing Instructions:
- Ask for option 1 "Membership fee". For variant 2 'contribution'.
- The euro amounts are queried randomly, so that one third of the surveys are available for each payment vector (both in variant 1 and in variant 2).

Suppose the monthly (member)contribution is **5/10/15 euros** per person. Would you be willing, taking into account your income situation, to pay this ***(member)***contribution to the maintenance of the mobile home hospice teams?

DO NOT READ!
1: Yes → **Continue to question 13a**

The original validated version of this questionnaire is in German. This translation into English was created with the translation tool of the European Commission eTranslation version 13.4 (https://webgate.ec.europa.eu/etranslation/about.html) and is not validated.
All rights are reserved to the authors in accordance with applicable copyright and intellectual property laws. Any unauthorized use or reproduction of this questionnaire will result in legal action.

Content refers to: Steigenberger, C., Leiter, A.M., Siebert, U., Schusterschitz, C., Flatscher-Thoeni, M. (2025). Influence of prior knowledge and experience on willingness to pay for home hospice services: a contingent valuation study. *International Journal of Health Economics and Management.* DOI: 10.1007/s10754-025-09393-8
Requests related to this questionnaire can be sent to andrea.leiter@uibk.ac.at.

2: No → **Continue to question 13b**
3: Don't know → **Continue to question 13b**
4: Not specified → **Continue to question 13b**

13a.
What if the monthly **(member)** contribution was **10/20/30 euros** per person? Would you be willing to pay this **(membership)** contribution?

DO NOT READ!
1: Yes → **Continue to question 14**
2: No → **Continue to question 14**
3: Don't know → **Continue to question 14**
4: Not specified → **Continue to question 14**

13b.
What if the monthly ***(member)*** contribution was **2.50/5/7.50 euros** per person? Would you be willing to pay this ***(membership)*** contribution?

DO NOT READ!
1: Yes → **Continue to question 14**
2: No → **Continue to question 13c**
3: Don't know → **Continue to question 13c**
4: Not specified → **Continue to question 13c**

13c.
Would you generally be willing to pay anything to maintain mobile home hospice teams?

DO NOT READ!
1: Yes → **Continue to question 13d**
2: No → **Continue to question 15**
3: Don't know → **Continue to question 15**
4: Not specified → **Continue to question 15**

13d.
INTERVIEWER: If the respondent mentions an amount spontaneously, please note.
Write down the amount in euros and cents!

_____ euros ______ cent

9998: Spontaneously no amount mentioned
9999: Don't know/No information

All rights are reserved to the authors in accordance with applicable copyright and intellectual property laws. Any unauthorized use or reproduction of this questionnaire will result in legal action.

Content refers to: Steigenberger, C., Leiter, A.M., Siebert, U., Schusterschitz, C., Flatscher-Thoeni, M. (2025). Influence of prior knowledge and experience on willingness to pay for home hospice services: a contingent valuation study. *International Journal of Health Economics and Management.* DOI: 10.1007/s10754-025-09393-8
Requests related to this questionnaire can be sent to andrea.leiter@uibk.ac.at.

14.
I now read you various reasons that can speak for the payment of a financial contribution. Please tell me about each statement, is that a reason why you agreed to pay a financial contribution?

DO NOT READ!
1: Yes
2: No
3: Don't know
4: Not specified

READ!
1: to be able to claim the benefits for myself in case of need
ITEM 2 ONLY ON VARIANT 1 REQUEST!
2: Because membership entails social recognition
3: Because I find the hospice facilities worth supporting
4: Because it's good to know that there are hospice facilities
ITEM 5 ONLY FOR VARIANT 1 REQUEST!
5: Because I'm proud to be a member of the hospice
6: Because I want to ensure the future provision of hospice facilities to society
POINT 7 ONLY FOR VARIANT 2 REQUEST!
7: So that my family can receive support when needed

→Continue to question 16

15.
I now read to you various reasons that can speak against the payment of a financial contribution. For each statement, please tell me if this is a reason why you did not agree to pay a financial contribution?

DO NOT READ!
1: Yes
2: No
3: Don't know
4: Not specified

READ!
1: Because the amounts are too high
2: Because such services have to be financed by the public sector
3: Because I don't see a need for hospice services for me

POINT 4 ONLY ON VARIANT 2 REQUEST!
4: Because everyone should finance such services themselves

The original validated version of this questionnaire is in German. This translation into English was created with the translation tool of the European Commission eTranslation version 13.4 (https://webgate.ec.europa.eu/etranslation/about.html) and is not validated.
All rights are reserved to the authors in accordance with applicable copyright and intellectual property laws. Any unauthorized use or reproduction of this questionnaire will result in legal action.

Content refers to: Steigenberger, C., Leiter, A.M., Siebert, U., Schusterschitz, C., Flatscher-Thoeni, M. (2025). Influence of prior knowledge and experience on willingness to pay for home hospice services: a contingent valuation study. *International Journal of Health Economics and Management.* DOI: 10.1007/s10754-025-09393-8
Requests related to this questionnaire can be sent to andrea.leiter@uibk.ac.at.

16. ***ONLY WITH VARIANT 1 REQUEST!***
Would depend on your willingness to provide a (IT: If the answer to question 13 to 13c is 'no' 3 times: If for humanitarian reasons non-members can also benefit from the home hospice services if necessary, do they have to pay a membership fee?

READ!
1: No, wouldn't change → **Continue to question 17**
2: Yes, I would be willing to pay more → **Continue to question 16a**
3: Yes, but I would pay less → **Continue to question 16b**
DO NOT READ!
4: Don't know → **Continue to question 17**
5: Not specified → **Continue to question 17**

16a. ***ONLY WITH VARIANT 1 REQUEST!***
How much would you be willing to pay **more per month?**

_______ euros → **Continue to question 17**

DO NOT READ!
88: Don't know
99: Not specified

16b. ***ONLY WITH VARIANTE 1 REQUEST!***
How much would you pay **less per month?**

_____ euros

DO NOT READ!
88: Don't know
99: Not specified

17.
Now on to something else: May I ask you which religious community you belong to?

DO NOT READ!
1: Roman Catholic Church
2: Protestant Church
3: Orthodox Church
4: Judaism
5: Islam
6: Hinduism

The original validated version of this questionnaire is in German. This translation into English was created with the translation tool of the European Commission eTranslation version 13.4 (https://webgate.ec.europa.eu/etranslation/about.html) and is not validated.
All rights are reserved to the authors in accordance with applicable copyright and intellectual property laws. Any unauthorized use or reproduction of this questionnaire will result in legal action.

Content refers to: Steigenberger, C., Leiter, A.M., Siebert, U., Schusterschitz, C., Flatscher-Thoeni, M. (2025). Influence of prior knowledge and experience on willingness to pay for home hospice services: a contingent valuation study. *International Journal of Health Economics and Management.* DOI: 10.1007/s10754-025-09393-8
Requests related to this questionnaire can be sent to andrea.leiter@uibk.ac.at.

7: Buddhism
8: Other religious community
9: Do not belong to any religious community
10: Not specified

18.
Apart from weddings, funerals and baptisms: How often do you go to church or similar religious gatherings?

DO NOT READ!
1: Several times a week
2: Once a week
3: About once a month
4: About once a year
5: Rare
6: Only on special holidays
7: Never go to religious gatherings.
8: Not specified

19.
No matter how often you attend church services or similar religious gatherings: Would you say you are:

READ!
1: A believing person
2: Not a believer
DO NOT READ!
3: Not specified

20.
What role does God play in your life? Tell me this on a scale of 1 to 6. 1 means God is not important at all in your life, 6 means very important. In between, you can graduate.

DO NOT READ!
1: Not important at all (1)
2:
3:
4:
5:
6: Very important (6)
7: Not specified

The original validated version of this questionnaire is in German. This translation into English was created with the translation tool of the European Commission eTranslation version 13.4 (https://webgate.ec.europa.eu/etranslation/about.html) and is not validated.
All rights are reserved to the authors in accordance with applicable copyright and intellectual property laws. Any unauthorized use or reproduction of this questionnaire will result in legal action.

Content refers to: Steigenberger, C., Leiter, A.M., Siebert, U., Schusterschitz, C., Flatscher-Thoeni, M. (2025). Influence of prior knowledge and experience on willingness to pay for home hospice services: a contingent valuation study. *International Journal of Health Economics and Management.* DOI: 10.1007/s10754-025-09393-8
Requests related to this questionnaire can be sent to andrea.leiter@uibk.ac.at.

21.
To what extent are your daily actions determined by your religious beliefs? Tell me this on a scale of 1 to 6. 1 means your daily actions are not determined by your religious beliefs at all, 6 means very strongly determined. In between, you can graduate.

DO NOT READ!
1: Not at all (1)
2:
3:
4:
5:
6: Very strong (6)
7: Not specified

22.
May I ask you, have you ever seen someone die?

READ!
MULTIPLE REPLY POSSIBLE! ***(except code 1 – here only single reply!)***
1: never before
2: Yes, because of my profession.
3: Yes, in the context of a voluntary activity
4: Yes, from my private environment.
DO NOT READ!
5: Not specified

23.
How many people close to you have died in the last 5 years?

_______ Number of persons

DO NOT READ!
99: Not specified

24.
I am now reading various statements to you. Tell me to everyone whether you agree or disagree.

DO NOT READ!
1: Yes, agree
2: No, don't agree
3: Don't know
4: Not specified

The original validated version of this questionnaire is in German. This translation into English was created with the translation tool of the European Commission eTranslation version 13.4 (https://webgate.ec.europa.eu/etranslation/about.html) and is not validated.
All rights are reserved to the authors in accordance with applicable copyright and intellectual property laws. Any unauthorized use or reproduction of this questionnaire will result in legal action.

Content refers to: Steigenberger, C., Leiter, A.M., Siebert, U., Schusterschitz, C., Flatscher-Thoeni, M. (2025). Influence of prior knowledge and experience on willingness to pay for home hospice services: a contingent valuation study. *International Journal of Health Economics and Management.* DOI: 10.1007/s10754-025-09393-8
Requests related to this questionnaire can be sent to andrea.leiter@uibk.ac.at.

READ!
a) Death is part of life
b) I believe in life after death
c) I think a lot about dying and death
d) I don't like to think about dying and death
e) Dignified dying is important to me
f) Active euthanasia is an acceptable course of action
g) Not dying alone is important to me
h) Every person should have the freedom to put an end to his or her own life
i) The prospect of my death frightens me
j) It would burden me to have to spend a lot of time with the dying

---

25.
Briefly on the subject of elections: Think back to the last national election. This National Council election was held on 28 September 2008. Did you vote in this last national election?

DO NOT READ!
1: Yes
2: No
3: Not specified

26.
If it were a national election tomorrow, which party would you vote for?

DO NOT READ!
1: SPÖ
2: ÖVP
3: FPÖ
4: Die Grünen
5: BZÖ
6: KPÖ
7: Other party

(1–7) → **Continue to question 27**

8: None, I would not vote → **Continue to question S3**
9: No answer → **Continue to question S3**

27.
Which party would be your second choice?

DO NOT READ!
1: SPÖ
2: ÖVP
3: FPÖ
4: Die Grünen

The original validated version of this questionnaire is in German. This translation into English was created with the translation tool of the European Commission eTranslation version 13.4 (https://webgate.ec.europa.eu/etranslation/about.html) and is not validated.
All rights are reserved to the authors in accordance with applicable copyright and intellectual property laws. Any unauthorized use or reproduction of this questionnaire will result in legal action.

Content refers to: Steigenberger, C., Leiter, A.M., Siebert, U., Schusterschitz, C., Flatscher-Thoeni, M. (2025). Influence of prior knowledge and experience on willingness to pay for home hospice services: a contingent valuation study. *International Journal of Health Economics and Management.* DOI: 10.1007/s10754-025-09393-8
Requests related to this questionnaire can be sent to andrea.leiter@uibk.ac.at.

5: BZÖ
6: KPÖ
7: Other party
8: I don't have one, I don't have a second choice
9: Not specified

Finally, may I ask you for some statistical information?

S3.
May I ask you about the size of your place of residence?

DO NOT READ!
1: Less than 2,000 inhabitants
2: 2,000 - 5,000 inhabitants
3: 5,001 - 10,000 inhabitants
4: 10,001 - 30,000 inhabitants
5: 30,001 - 100,000 inhabitants
6: Over 100,000 inhabitants
7: Don't know

S4.
In which state do you live?

DO NOT READ!
1: Burgenland
2: Carinthia
3: Lower Austria
4: Upper Austria
5: Salzburg
6: Styria
7: Tyrol
8: Vorarlberg
9: Vienna

S5.
In which political district do you live?

DO NOT READ!
SELECT FROM LIST OF POLITICAL DISTRICTS

The original validated version of this questionnaire is in German. This translation into English was created with the translation tool of the European Commission eTranslation version 13.4 (https://webgate.ec.europa.eu/etranslation/about.html) and is not validated.
All rights are reserved to the authors in accordance with applicable copyright and intellectual property laws. Any unauthorized use or reproduction of this questionnaire will result in legal action.

Content refers to: Steigenberger, C., Leiter, A.M., Siebert, U., Schusterschitz, C., Flatscher-Thoeni, M. (2025). Influence of prior knowledge and experience on willingness to pay for home hospice services: a contingent valuation study. *International Journal of Health Economics and Management.* DOI: 10.1007/s10754-025-09393-8
Requests related to this questionnaire can be sent to andrea.leiter@uibk.ac.at.

S6.
How many years have you been living in your place of residence?
_________ years

DO NOT READ!
98: Don't know
99: Not specified

S7.
In which country were you born?

DO NOT READ!

EUROPE:
1: Austria
2: Albania
3: Armenia
4: Azerbaijan
5: Belarus
6: Belgium
7: Bosnia and Herzegovina
8: Bulgaria
9: Denmark
10: Germany
11: England
12: Estonia
13: Finland
14: France
15: Georgia
16: Greece
17: Ireland
18: Iceland
19: Italy
20: Kosovo
21: Croatia
22: Latvia
23: Liechtenstein
24: Lithuania
25: Luxembourg
26: Malta
27: Macedonia
28: Moldova
29: Montenegro
30: Netherlands
31: Norway

The original validated version of this questionnaire is in German. This translation into English was created with the translation tool of the European Commission eTranslation version 13.4 (https://webgate.ec.europa.eu/etranslation/about.html) and is not validated.
All rights are reserved to the authors in accordance with applicable copyright and intellectual property laws. Any unauthorized use or reproduction of this questionnaire will result in legal action.

Content refers to: Steigenberger, C., Leiter, A.M., Siebert, U., Schusterschitz, C., Flatscher-Thoeni, M. (2025). Influence of prior knowledge and experience on willingness to pay for home hospice services: a contingent valuation study. *International Journal of Health Economics and Management.* DOI: 10.1007/s10754-025-09393-8
Requests related to this questionnaire can be sent to andrea.leiter@uibk.ac.at.

32: Poland
33: Portugal
34: Romania
35: Russian Federation
36: Sweden
37: Switzerland
38: Serbia
39: Slovakia
40: Slovenia
41: Spain
42: Czech Republic
43: Turkey
44: Ukraine
45: Hungary
46: Cyprus

ASIA:
47: Bangladesh
48: China
49: India
50: Indonesia
51: Iraq
52: Iran
53: Israel
54: Japan
55: Jordan
56: Cambodia
57: Korea
58: Lebanon
59: Pakistan
60: Palestine
61: Philippines
62: Saudi Arabia
63: Syria
64: Taiwan
65: Thailand
66: Vietnam
67: Other countries of the Middle East
68: Other Asian countries

AMERICA:
69: Argentina
70: Brazil
71: Chile
72: Canada

The original validated version of this questionnaire is in German. This translation into English was created with the translation tool of the European Commission eTranslation version 13.4 (https://webgate.ec.europa.eu/etranslation/about.html) and is not validated.
All rights are reserved to the authors in accordance with applicable copyright and intellectual property laws. Any unauthorized use or reproduction of this questionnaire will result in legal action.

Content refers to: Steigenberger, C., Leiter, A.M., Siebert, U., Schusterschitz, C., Flatscher-Thoeni, M. (2025). Influence of prior knowledge and experience on willingness to pay for home hospice services: a contingent valuation study. *International Journal of Health Economics and Management.* DOI: 10.1007/s10754-025-09393-8
Requests related to this questionnaire can be sent to andrea.leiter@uibk.ac.at.

73: Mexico
74: Peru
75: Other Central/South American countries
76: United States

AFRICA:
77: Egypt
78: Algeria
79: Libya
80: Morocco
81: Nigeria
82: Republic of South Africa
83: Sudan
84: Tunisia
85: Other African States

86: Australia and Ozenania
87: Other State
88: Not specified

S8.
What nationality do you have?

DO NOT READ!
1: Austria
2: Bosnia and Herzegovina
3: Germany
4: Italy
5: Kosovo
6: Croatia
7: Macedonia
8: Montenegro
9: Poland
10: Romania
11: Switzerland
12: Serbia
13: Slovakia
14: Slovenia
15: Czech Republic
16: Turkey
17: Hungary
18: United States
19: Other EU country
20: Other state
21: Not specified

The original validated version of this questionnaire is in German. This translation into English was created with the translation tool of the European Commission eTranslation version 13.4 (https://webgate.ec.europa.eu/etranslation/about.html) and is not validated.
All rights are reserved to the authors in accordance with applicable copyright and intellectual property laws. Any unauthorized use or reproduction of this questionnaire will result in legal action.

Content refers to: Steigenberger, C., Leiter, A.M., Siebert, U., Schusterschitz, C., Flatscher-Thoeni, M. (2025). Influence of prior knowledge and experience on willingness to pay for home hospice services: a contingent valuation study. *International Journal of Health Economics and Management.* DOI: 10.1007/s10754-025-09393-8
Requests related to this questionnaire can be sent to andrea.leiter@uibk.ac.at.

S9.
What is your native language?

DO NOT READ!
1: German
2: Albanian
3: Arabic
4: Bosnian
5: Chinese
6: English
7: French
8: Italian
9: Croatian
10: Kurdish
11: Macedonian
12: Persian
13: Polish
14: Romanian
15: Serbian
16: Slovak
17: Slovenian
18: Czech
19: Turkish
20: Hungarian
21: Other European languages
22: Other African languages
23: Other Asian languages
24: Other languages
25: Not specified

S10.
How would you describe your state of health in general?

READ!
1: Excellent (1)
2: Very good (2)
3: Good (3)
4: Less good (4)
5: Bad (5)
DO NOT READ!
6: Not specified

The original validated version of this questionnaire is in German. This translation into English was created with the translation tool of the European Commission eTranslation version 13.4 (https://webgate.ec.europa.eu/etranslation/about.html) and is not validated.
All rights are reserved to the authors in accordance with applicable copyright and intellectual property laws. Any unauthorized use or reproduction of this questionnaire will result in legal action.

Content refers to: Steigenberger, C., Leiter, A.M., Siebert, U., Schusterschitz, C., Flatscher-Thoeni, M. (2025). Influence of prior knowledge and experience on willingness to pay for home hospice services: a contingent valuation study. *International Journal of Health Economics and Management.* DOI: 10.1007/s10754-025-09393-8
Requests related to this questionnaire can be sent to andrea.leiter@uibk.ac.at.

S12.
How do you assess your health compared to other people of your age?

READ!
1: Excellent (1)
2: Very good (2)
3: Good (3)
4: Less good (4)
5: Bad (5)
DO NOT READ!
6: Not specified

S13.
Have you ever been so sick that you were close to death?

DO NOT READ!
1: Yes
2: No
3: Not specified

S14. **AGE FILTERS - only for men aged 60 years or younger or only for women aged 65 years or younger.**
Based on your overall health, do you think you will reach the average age of 78 for men and 83 for women?

DO NOT READ!
1: Yes
2: No
3: Not specified

S15.
May I ask you about your marital status? Are you-

READ!
1: Single → **Continue to question S17**
2: Married → **Continue to question S16**
3: Life partnership → **Continue to question S16**
4: Divorced → **Continue to question S17**
5: Widowed → **Continue to question S17**
DO NOT READ!
6: Not specified → **Continue to question S17**

The original validated version of this questionnaire is in German. This translation into English was created with the translation tool of the European Commission eTranslation version 13.4 (https://webgate.ec.europa.eu/etranslation/about.html) and is not validated.
All rights are reserved to the authors in accordance with applicable copyright and intellectual property laws. Any unauthorized use or reproduction of this questionnaire will result in legal action.

Content refers to: Steigenberger, C., Leiter, A.M., Siebert, U., Schusterschitz, C., Flatscher-Thoeni, M. (2025). Influence of prior knowledge and experience on willingness to pay for home hospice services: a contingent valuation study. *International Journal of Health Economics and Management.* DOI: 10.1007/s10754-025-09393-8
Requests related to this questionnaire can be sent to andrea.leiter@uibk.ac.at.

S16. ***ONLY REQUEST IF NOT SPECIFIED CODE 3 IN QUESTION 4!***
May I ask you how old your partner is?

S17.
How many people, including yourself, live in your household?

S18. ***ALSO REQUEST, IF 1 IN QUESTION S17!***
May I ask you about the number of your children under the age of 18?

a) Number of biological children under the age of 18: ______
b) Number of non-biological children under the age of 18: _______

S19.
How many children (including those under the age of 18) do you provide with your income?

S21.
How many people in your household receive an income?

S22.
What is the highest level of education you have completed?

DO NOT READ!
1: No compulsory school leaving certificate
2: Compulsory school leaving certificate (Volks- und Hauptschule)
3: Apprenticeship qualification (vocational school)
4: Completion of a vocational middle school / technical school (e.g., commercial school)
5: Matura (Professional or General Higher Education)
6: Further education, e.g., college, university course
7: Completion of a technical school or university degree
8: Not specified

The original validated version of this questionnaire is in German. This translation into English was created with the translation tool of the European Commission eTranslation version 13.4 (https://webgate.ec.europa.eu/etranslation/about.html) and is not validated.
All rights are reserved to the authors in accordance with applicable copyright and intellectual property laws. Any unauthorized use or reproduction of this questionnaire will result in legal action.

Content refers to: Steigenberger, C., Leiter, A.M., Siebert, U., Schusterschitz, C., Flatscher-Thoeni, M. (2025). Influence of prior knowledge and experience on willingness to pay for home hospice services: a contingent valuation study. *International Journal of Health Economics and Management.* DOI: 10.1007/s10754-025-09393-8
Requests related to this questionnaire can be sent to andrea.leiter@uibk.ac.at.

S23.
Are you currently working?

DO NOT READ!
1: Yes → **Continue to question S23a**
2: No → **Continue to question S24**
3: Not specified → **Continue to question S26**

S23a.
Are you –

READ!
1: Full-time employment, i.e., more than 34 hours per week
2: Working part-time, i.e., more than 15-34 hours per week
3: Slightly employed, i.e., less than 15 hours per week

S23b.
Are you –

READ!
1: apprentice
2: worker
3: clerk
4: Civil servant
5: Self-employed
6: Celebration of Employees
7: farmer
8: in parental leave
DO NOT READ!
9: Not specified

S23c.
Do you have a managerial or executive role?

DO NOT READ!
1: Managerial position → **Continue to question S26**
2: Executive position → **Continue to question S26**
3: Not specified → **Continue to question S26**

The original validated version of this questionnaire is in German. This translation into English was created with the translation tool of the European Commission eTranslation version 13.4 (https://webgate.ec.europa.eu/etranslation/about.html) and is not validated.
All rights are reserved to the authors in accordance with applicable copyright and intellectual property laws. Any unauthorized use or reproduction of this questionnaire will result in legal action.

Content refers to: Steigenberger, C., Leiter, A.M., Siebert, U., Schusterschitz, C., Flatscher-Thoeni, M. (2025). Influence of prior knowledge and experience on willingness to pay for home hospice services: a contingent valuation study. *International Journal of Health Economics and Management.* DOI: 10.1007/s10754-025-09393-8
Requests related to this questionnaire can be sent to andrea.leiter@uibk.ac.at.

S24.
Are you –

READ!
1: In training
2: Housewife/housekeeper
3: Unemployed
4: Pensioner
DO NOT READ!
5: Not specified

S26.
Do you care for a person in need of care living in your household, e.g., partner, parents, or other relatives?

DO NOT READ!
1: Yes
2: No
3: Not specified

S27.
And do you care for or care for people in need of care outside of your household?
DO NOT READ!

1: Yes
2: No
3: Not specified

S28.
May I ask you which sector of the economy you are in, or have you been working full-time (if you are retired)?

DO NOT READ! ONLY ACCORDING!
1: Agriculture and forestry, including fishing and fish farming
2: Mining and quarrying
3: Manufacturing, manufacturing of goods, e.g., manufacturing of clothing, food, furniture, mechanical engineering, etc.
4: Energy supply, e.g., storm and gas supply
5: Water supply, sewage and waste management and remediation of pollution
6: Construction including construction installation
7: Retail or wholesale trade; Maintenance and repair of motor vehicles
8: Hospitality, accommodation and gastronomy, tourism
9: Transport and storage, including passenger transport, freight transport, aviation, postal, courier and express services

The original validated version of this questionnaire is in German. This translation into English was created with the translation tool of the European Commission eTranslation version 13.4 (https://webgate.ec.europa.eu/etranslation/about.html) and is not validated.
All rights are reserved to the authors in accordance with applicable copyright and intellectual property laws. Any unauthorized use or reproduction of this questionnaire will result in legal action.

Content refers to: Steigenberger, C., Leiter, A.M., Siebert, U., Schusterschitz, C., Flatscher-Thoeni, M. (2025). Influence of prior knowledge and experience on willingness to pay for home hospice services: a contingent valuation study. *International Journal of Health Economics and Management.* DOI: 10.1007/s10754-025-09393-8
Requests related to this questionnaire can be sent to andrea.leiter@uibk.ac.at.

10: Information and communication, including publishing, broadcasting, news, telecommunications, data processing
11: Provision of financial and insurance services
12: Land and housing
13: Provision of professional, scientific, technical services, e.g., legal advice, architectural office, advertising, research, photography
14: Provision of other economic services, e.g., video library, rental of objects, travel agency, security and security service, caretaker, cleaning of buildings, etc.
14: Public administration, defence, social security
15: Education
16: Health, Veterinary, and Social Services
17: Art, entertainment, and recreation
18: Provision of other services, e.g., interest groups as well as church and other associations
19: Households with domestic staff, production of goods and services by households for own use
20: Extraterritorial organizations and bodies, e.g., activities of international organizations, diplomatic representation
21: I've never been working
22: Not specified

S29.
If you add up all available salaries, pensions, unemployed, etc., your monthly household net income, i.e., the income after deduction of taxes, is:

READ!
1: less than 500 euros
2: 500 - 999 euros
3: 1,000 – 1,499 euros
4: 1,500 – 1,999 euros
5: 2,000 – 2,499 euros
6: 2,500 – 2,999 euros
7: 3,000 – 3,499 euros
8: 3,500 – 3,999 euros
9: 4,000 – 4,999 euros
10: 5,000 euros and more

} **Continue to question S30**

DO NOT READ!
11: Not specified → **Continue to question S31**

The original validated version of this questionnaire is in German. This translation into English was created with the translation tool of the European Commission eTranslation version 13.4 (https://webgate.ec.europa.eu/etranslation/about.html) and is not validated.
All rights are reserved to the authors in accordance with applicable copyright and intellectual property laws. Any unauthorized use or reproduction of this questionnaire will result in legal action.

Content refers to: Steigenberger, C., Leiter, A.M., Siebert, U., Schusterschitz, C., Flatscher-Thoeni, M. (2025). Influence of prior knowledge and experience on willingness to pay for home hospice services: a contingent valuation study. *International Journal of Health Economics and Management.* DOI: 10.1007/s10754-025-09393-8
Requests related to this questionnaire can be sent to andrea.leiter@uibk.ac.at.

S30.
What does net monthly household income include?

READ!
MULTIPLE REPLY POSSIBLE!

1: Own income
2: Income of other persons living in the household
3: Financial contributions from persons not living in the household
4: Social benefits (child, social allowance, care allowance, rent allowance, etc.)
5: Other income
DO NOT READ!
6: Not specified

S31.
May I ask you for your personal monthly net income? If your current personal monthly net income is currently -

READ!
1: Currently no income
2: Less than 200 euros
3: 200 - 499 euros
4: 500 - 999 euros
5: 1,000 - 1,499 euros
6: 1,500 - 1,999 euros
7: 2,000 - 2,499 euros
8: 2,500 - 2,999 euros
9: 3,000 - 3,499 euros
10: 3,500 - 3,999 euros
11: 4,000 - 4,999 euros
12: 5,000 euros and more
DO NOT READ!
13: Not specified

Thank you for the interview and for taking your time. I wish you a nice day/evening.

The original validated version of this questionnaire is in German. This translation into English was created with the translation tool of the European Commission eTranslation version 13.4 (https://webgate.ec.europa.eu/etranslation/about.html) and is not validated.
All rights are reserved to the authors in accordance with applicable copyright and intellectual property laws. Any unauthorized use or reproduction of this questionnaire will result in legal action.
